# Manipulating the Migration of Iodine Ions via Reverse‐Biasing for Boosting Photovoltaic Performance of Perovskite Solar Cells

**DOI:** 10.1002/advs.202204163

**Published:** 2022-10-26

**Authors:** Keqing Huang, Xiangxiang Feng, Hengyue Li, Caoyu Long, Biao Liu, Jiangjian Shi, Qingbo Meng, Klaus Weber, The Duong, Junliang Yang

**Affiliations:** ^1^ Hunan Key Laboratory for Super‐Microstructure and Ultrafast Process School of Physics and Electronics Central South University Changsha 410083 China; ^2^ College of Engineering and Computer Science Australian National University Canberra Australian Capital Territory Canberra 2600 Australia; ^3^ Key Laboratory for Renewable Energy Chinese Academy of Sciences Beijing Key Laboratory for New Energy Materials and Devices Institute of Physics Chinese Academy of Sciences Beijing 100190 P. R. China

**Keywords:** ion migration, perovskite solar cells, reverse bias, vacancy

## Abstract

Perovskite solar cells (PSCs) are being developed rapidly and exhibit greatly potential commercialization. Herein, it is found that the device performance can be improved by manipulating the migration of iodine ions via reverse‐biasing, for example, at −0.4 V for 3 min in dark. Characterizations suggest that reverse bias can increase the charge recombination resistance, improve carrier transport, and enhance built‐in electric field. Iodine ions including iodine interstitials in perovskites are confirmed to migrate and accumulate at the SnO_2_/perovskite interface under reverse‐basing, which fill iodine vacancies at the interface and interact with SnO_2_. First‐principles calculations suggest that the SnO_2_/perovskite interface with less iodine vacancies has a stronger interaction and higher charge transfer, leading to larger built‐in electric field and improved charge transport. Iodine ions that may pass through the SnO_2_/perovskite interface are also confirmed to be able to interact with Sn^4+^ and passivate oxygen vacancies on the surface of SnO_2_. Consequently, an efficiency of 23.48% with the open‐circuit voltage (*V*_oc_) of 1.16 V is achieved for PSCs with reverse‐biasing, as compared with the initial efficiency of 22.13% with a *V*_oc_ of 1.10 V. These results are of great significance to reveal the physics mechanism of PSCs under electric field.

## Graphical abstract

Performance of perovskitesolar cells can be improved by reverse‐biasing. Characterizations suggest reverse bias can increasecharge recombination resistance and improve carrier transport. It should beattributed to iodine ion migration, which reduces interfacial iodine vacanciesand passivates oxygen vacancies on tin dioxide. Consequently, an efficiency of 23.48% with the open‐circuit voltage of 1.16 V is achieved.

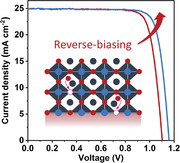

## 1 Introduction

Perovskite solar cells (PSCs) are being developed rapidly during the past decade and the power conversion efficiency (PCE) as high as 25.7% has been achieved.^[^ ^1^, ^2^, ^3^ ^]^ Because of the advantages of high efficiency, solution processability, and light weight,^[^ ^4^, ^5^ ^]^ PSCs exhibit great application potentials in the field of photovoltaics. However, perovskites and PSCs still face the problem of instability. Apart from oxygen and humidity that can be excluded by encapsulation, intrinsic instability of perovskites under light, heat stimuli and electrical bias, and the ion migration inside the device are more critical.

In actual fields, the module shaded by external factors such as birds and snow has to bear the reverse bias created by other parts exposed to light. This will cause a series of problems for PSCs. For example, McGehee and co‐workers found that reverse bias could cause a partially recoverable loss in PCE and open‐circuit voltage (*V*_oc_).^[^ ^6^ ^]^ Jeangros and co‐workers further found that different values of reverse voltage would bring various problems to PSCs, including the formation of highly conductive shunts in active area, ion migration from the perovskite absorber to the charge transport layer, and irreversible degradation of the perovskite layer into iodide‐ and bromide‐rich sub‐layers.^[^ ^7^ ^]^ Also, it has been confirmed that the tunneling holes into the perovskite due to sharp band bending can trigger the oxidation of halides to form neutral halogens, which act as bulk recombination centers.^[^ ^8^ ^]^ Recently, the interaction between the iodide interstitials and injected holes at the interface between perovskite and electron transport layer (ETL), which triggers the degradation of PSCs under reverse bias, has been confirmed by drive‐level capacitance profiling technique.^[^ ^9^ ^]^ Considering that the current density–voltage (*J–V*) hysteresis in PSCs is generally attributed to the carrier accumulation and defects at the interfaces,^[^ ^10^ ^]^ the ion migration induced by external stimuli can also affect the hysteresis. For instance, Jen and co‐workers found that reverse bias could result in abnormal hysteresis, which is similar to tunnel diode current–voltage characteristics.^[^ ^11^ ^]^

Nonetheless, ion movement and charge transfer in perovskite or devices are not always harmful, especially when the process is controlled within a recoverable range. For example, continuous tuning of the work function and varying the polarity of formamidinium lead triiodide (FAPbI_3_) perovskite films could be realized by the negative and positive applied voltages.^[^ ^12^ ^]^ Also, n‐doped CsPbBr_3_ nanocrystal thin films could be achieved via electrochemical doping, leading to an increased photoluminescence intensity.^[^ ^13^ ^]^ Huang and co‐workers found that the efficiency of PSCs can increase and partially recover during gamma‐ray radiation, which would be closely related to the self‐healing induced by ion‐migration behavior.^[^ ^14^ ^]^ Meng and co‐workers found the improved device performance during maximum power point tracking (MPPT) or under forward bias, which was attributed to the filled traps by moving ions during the operation.^[^ ^15^ ^]^ Generally, the dark recovery phenomenon after continuous illumination and MPPT should also be closely related to the ion migration in the perovskite layer.^[^ ^16^, ^17^, ^18^ ^]^ Very recently, Choi and co‐workers applied a pulsatile reverse pulse (30 s) in the middle of MPPT to eliminate accumulated charges and ions, which can revive the degraded PSC and improve the stability of the working device.^[^ ^19^ ^]^ These results above provide a possibility that ion migration can be utilized via external stimuli to improve the performance and stability of PSCs.

Herein, it is the first time to find that the photovoltaic performance of the PSCs can be improved by reverse bias. Specifically, the *V*_oc_ can be improved with an average increment of 30 mV, while short‐circuit current density (*J*_sc_) and fill factor (FF) remain constant. Electrical characterizations, including Nyquist plots, Mott–Schottky curves and so on, also corroborated the positive effect of reverse bias. Additionally, it was found that halide ions in perovskite, especially iodine ions and iodine interstitials, can migrate and accumulate at the SnO_2_/perovskite interface under reverse bias. Interaction between halide ions and Sn^4+^ has been confirmed by X‐ray photoelectron spectroscopy (XPS) and first‐principles calculations, which causes a stronger interaction and higher charge transfer at the SnO_2_/perovskite interface. First‐principles calculations further confirm that the iodine ions possibly migrating into SnO_2_ layers can form strong Sn—I ionic bonds and passivate oxygen vacancies on the surface of SnO_2_. The search results are of great significance to utilize the ion migration manipulated via reverse bias for improving the photovoltaic performance and stability of PSCs.

## 2 Results and Discussion

**Figure 1:**
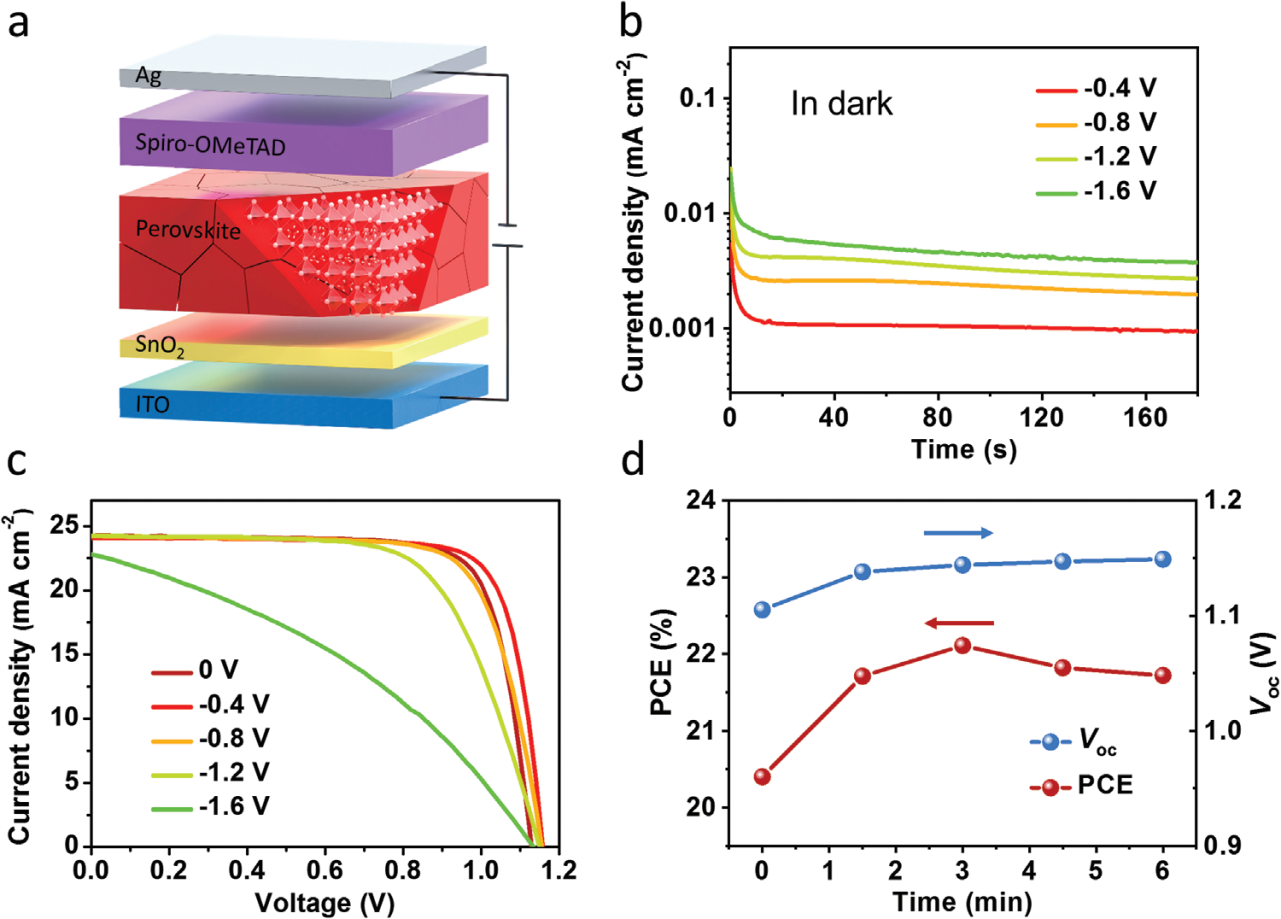
a) Schematic of the PSC under reverse bias, with a planar heterojunction structure of ITO/SnO_2_/Perovskite/Spiro‐OMeTAD/Ag. b) Monitoring of current densities of the PSC under different reverse bias voltages in dark. c) Reverse scanned *J–V* curves of the PSC after reverse‐biasing at different voltages for 3 min. d) PCE and *V*_oc_ of a PSC after reverse‐biasing at −0.4 V for the different time.

**Figure** **1**a shows the cell configuration in this work, namely ITO/SnO_2_/FA_0.945_MA_0.025_Cs_0.03_Pb(I_0.975_Br_0.025_)_3_/Spiro‐OMeTAD/Ag (ITO: indium tin oxide; Spiro‐OMeTAD: 2,2′,7,7′‐tetrakis(N,N‐dipmethoxyphenylamine)‐9,9′‐spirobifluorene), which is a typical high‐performance planar heterojunction PSC.^[^ ^20^, ^21^, ^22^, ^23^ ^]^ While the PSC is under reverse bias, the front ITO and rear Ag electrode contacts are connected to positive and negative terminals of external power supply, respectively. As shown in Figure 1b, the reverse current first decreases and then tends to be stable, which is consistent with the published result and will be discussed later.^[^ ^24^ ^]^ In addition, the current grows with the increase of the reverse voltage due to enhanced electronic and ionic currents under higher electric field. Moreover, it is found that the photovoltaic performance of PSCs is changed obviously after the devices are subjected to reverse bias for 3 min in dark. As shown in Figure 1c, the device performance is improved after the reverse‐biasing at −0.4 V for 3 min, mainly coming from the enhancement in *V*_oc_. Overall, the efficiency (*V*_oc_, *J*_sc_, and FF) of the PSC increases from the initial 20.50% (1.11 V, 24.24 mA cm^−2^, and 76.02%) to 21.95% (1.16 V, 24.09 mA cm^−2^, and 78.78%). *J–V* curves derived from forward scanning showed similar tendency (Figure S1, Supporting Information).

However, when the reverse bias exceeds −0.8 V, the device performance gradually decreases. This result is consistent with previous reports, in which ion migration, phase segregation, and the formation of highly conductive shunts due to silver melting or metal migration induced by reverse bias reduce the performance of PSCs.^[^ ^6^, ^7^ ^]^ The statistical results of device performance after reverse‐biasing at the different voltages further confirm the tendency above (**Table** **1**). Besides, the effect of loading time under reverse bias is investigated. As shown in Figure 1d, the *V*_oc_ first increases with the time under −0.4 V reverse bias and it becomes stable after 3 min. The efficiency also increases with the time under −0.4 V reverse bias, but it peaks at 3 min and then decreases over time. Although the changes in *J*_sc_ and FF are not remarkable during the whole period, prolonged reverse‐biasing results in a decrease in FF (Figure S2 and Table S1, Supporting Information). It should be attributed to the degradation of the perovskite triggered by ion migration under electric field.

**Table 1:** Performance data for PSCs before and after reverse‐biasing at different voltage for 3 min. The statistics are based on ten cells

Voltage [V]	*V*_oc_ [V])	*J*_sc_ [mA cm^−2^]	FF	PCE [%]	*R*_s_ [kΩ]	*R*_sh_ [kΩ]
0	1.12 ± 0.02	23.83 ± 0.37	0.764 ± 0.018	20.38 ± 0.56	0.04 ± 0.01	43.41 ± 29.33
−0.4	1.15 ± 0.01	23.84 ± 0.29	0.780 ± 0.011	21.41 ± 0.30	0.03 ± 0.01	127.37 ± 179.79
−0.8	1.14 ± 0.01	23.56 ± 0.46	0.688 ± 0.060	18.55 ± 1.92	0.10 ± 0.07	21.55 ± 10.38
−1.2	1.11 ± 0.05	22.90 ± 1.22	0.565 ± 0.097	14.48 ± 3.54	0.90 ± 1.22	31.28 ± 46.84
−1.6	1.07 ± 0.06	19.59 ± 2.08	0.359 ± 0.034	7.55 ± 1.32	4.83 ± 11.35	2.15 ± 1.22

As mentioned above, the negative effect of reverse bias on PSCs has been reported in the literature.^[^ ^6^, ^7^ ^]^ However, in this work, the positive effect of reverse bias is discovered for the first time. Therefore, this work will mainly focus on the unique and interesting phenomenon and try to explore the potential mechanism. Considering that the device performance can be greatly improved by reverse‐biasing at −0.4 V for 3 min, these parameters for reverse‐biasing are adopted in subsequent parts, unless otherwise specified.

Accordingly, a series of characterizations were carried out. Given the vital role of the perovskite absorber in PSCs, the perovskite layers before and after reverse‐biasing were characterized. It is found that the scanning electron microscopy (SEM) images, absorption spectra, and X‐ray diffraction (XRD) patterns of the perovskite layers are barely changed after reverse‐biasing at ‐0.4 V for 3 min (Figures S3–S5, Supporting Information). These results suggest that the relatively low reverse voltage does not deteriorate the surface morphology, absorbance, and crystal lattice structure of perovskite. SEM cross‐section images of the PSCs indicate that reverse bias has negligible effect on the stack structure and each layer of the cells as well (Figure S6, Supporting Information).

**Figure 2:**
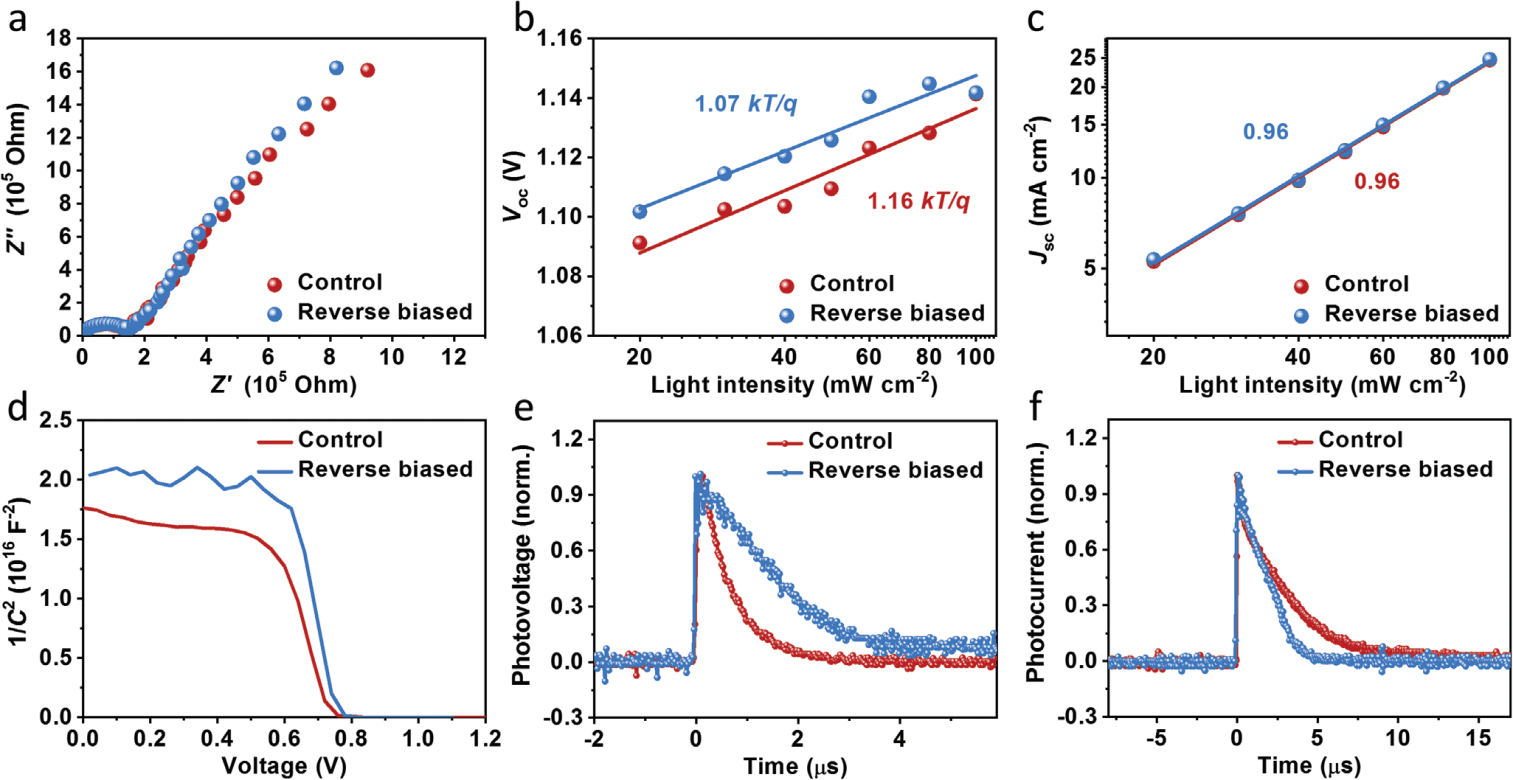
a) Nyquist plots of the PSC before and after reverse‐biasing at −0.4 V for 3 min. The light‐intensity dependence of b) *V*_oc_ and c) *J*_sc_ measurement related to pristine and reverse biased PSCs. d) Mott–Schottky curves, (e) transient photovoltage decay curves, and f) transient photocurrent decay curves of the PSCs before and after reverse‐biasing.

Electrochemical impedance spectroscopy (EIS) is employed to study carrier dynamics in PSCs. As shown in **Figure** **2**a, recombination resistance (*R*_rec_) assigned to low‐frequency increased in PSCs after reverse‐biasing. It indicates that the charge carrier recombination within the cell is suppressed. Additionally, it is found that the charge transfer resistance (*R*_ct_) is hardly changed after reverse‐biasing. These results are consistent with the change in series resistance (*R*_s_) and shunting resistance (*R*_sh_) obtained from *J–V* curves (Table 1). Figure 2b shows *V*_oc_ versus light intensity of the PSC before and after reverse‐biasing. It suggests that the *V*_oc_ values increase obviously after reverse‐biasing, irrespective of the light intensity. More importantly, the deviation between the slope and the value of (*kT*/*q*) decreases significantly after reverse‐biasing, indicating that the trap‐assisted recombination is suppressed.^[^ ^25^ ^]^ In contrast, the slopes between *J*_sc_ and light intensity are almost identical and very close to 1 (Figure 2c), suggesting that the bimolecular recombination is negligible and not affected by the reverse bias.^[^ ^25^ ^]^

In addition, the Mott–Schottky curves are obtained in dark via capacitance–voltage measurement to reveal the mechanism for improved *V*_oc_ and efficiencies. As shown in Figure 2d, the built‐in field (*V*_bi_) of the reverse biased cell is much higher than its initial value, indicating an increased driving force for charge separation and extended depletion region for suppressing the carrier recombination. The larger slope indicates the decreased interfacial charge density, which has an inversely proportional relationship to the slope.^[^ ^26^, ^27^ ^]^ It should be attributed to the ion migration, improved energy level alignment and accordingly reduced interfacial defects, which will be discussed below. Besides, the transient photovoltage and photocurrent decays are measured at open and short circuit, respectively. It is apparent that the photovoltage decay is slowed down, but the decay of photocurrent is accelerated after reverse‐biasing (Figure 2e,f). It suggests that the recombination of charge carriers is suppressed, and the charge extraction and transport are improved.

The results above show that the reverse bias has negligible effect on each layer in PSCs, but improves the device performance and charge carrier dynamics. However, the mechanism for the improvement in device performance is still not clear. Given that perovskites are ionic materials, ion migration under the external electric field may change the energy band alignment between perovskite and charge transport layers,^[^ ^12^ ^]^ thus improving device performance. In order to confirm the hypothesis, a series of PSCs based on different structures have been fabricated and their photovoltaic performance was measured before and after reverse‐biasing at −0.4 V for 3 min. The results show that the composition of perovskite does not affect the change in device performance after reverse‐biasing (Table S2, Supporting Information). It indicates that device performance based on this cell configuration can be improved, regardless of the perovskite composition. In contrast, the device performance is deteriorated when [6,6]‐phenyl‐C61‐butyric acid methyl ester (PC_61_BM) is used as an electron transport layer or to modify SnO_2_. Furthermore, the deterioration of device performance is observed in electron transport layer free PSCs, while the efficiency is still improved in hole transport layer (HTL) free PSCs. These results suggest that the electron transport layer of SnO_2_ or SnO_2_/perovskite interface plays a vital role in the improvement of device performance.

**Figure 3:**
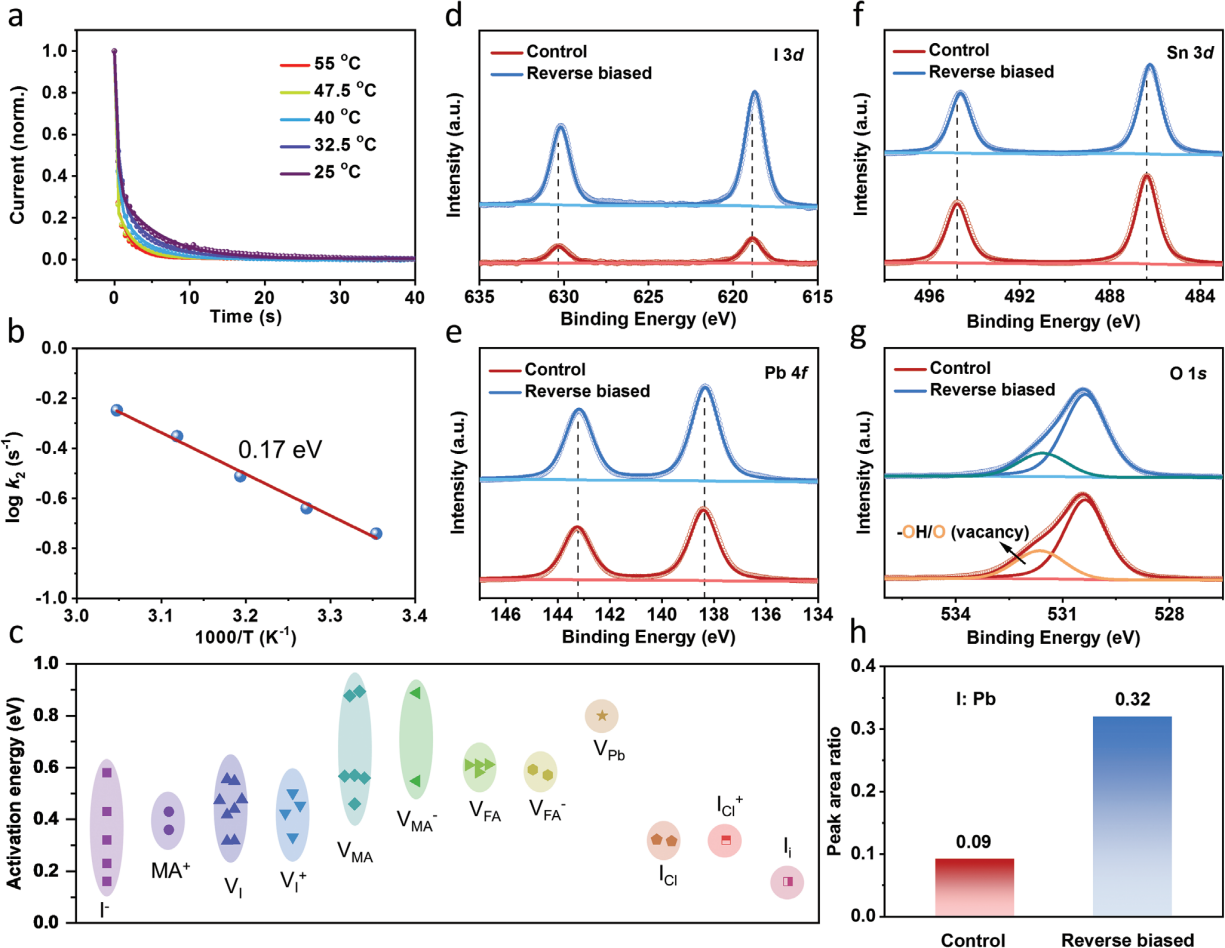
a) Current change of the PSCs under −0.4 V and different temperature in dark. b) The Arrhenius plot of the time constant. c) Activation energy of different type of ions in perovskite; XPS spectra of d) I 3d, e) Pb 4f, f) Sn 3d, and g) O 1s of SnO_2_ films. h) Peak area ratio between I 3d and Pb 4f of SnO_2_ films before and after reverse‐biasing at −0.4 V for 3 min.

**Figure** **3**a shows the current change of the PSCs under −0.4 V and different temperature in dark. They can be well fitted by the following equation

(1) $$I=I_{0}+I_{1}\exp\left(-k_{1}t\right)+I_{2}\exp\left(-k_{2}t\right)$$

The fitted parameters of the current–time (*I–t*) curves are shown in Table S3 in the Supporting Information. It has been reported that the capacitance of PSCs is very small (≈10^−2^ µF).^[^ ^28^ ^]^ Therefore, the rapid decrease at the beginning of the *I–t* curves should be attributed to the capacitive effect of PSCs, corresponding to the small value of *k*_1_. The following part of the slow decay should result from ion migration under reverse bias. Accordingly, the Arrhenius plot of current decay velocity *k*_2_ and the inverse time constant calculated from the *I–t* curves are shown in Figure 3b. The activation energy calculated from the slope of the curve is 0.17 eV. It only matches the activation energies of iodine ions and iodine interstitials, while the activation energy of other mobile ions in perovskites is much higher than this value (Figure 3c and Table S4, Supporting Information).^[^ ^29^, ^30^, ^31^ ^]^ It further confirms the speculation that iodine ion migration occurs in PSCs under reverse bias in dark.

Figure 3d–g shows the XPS spectra of the SnO_2_ layers before and after reverse‐biasing. It is noticed that the SnO_2_ films are derived from complete PSCs (see more details in the Experimental Section). The result shows that the intensity of I 3d peaks in reverse biased sample is much stronger than that of control sample, while the differences in the intensities of Pb 4f and Sn 3d peaks between the control and reverse‐biased sample are negligible, leading to the increase in the ratio of I:Pb (Figure 3h). It is assumed that I 3d and Pb 4f peaks in the control sample should be attributed to residual perovskite or lead iodide. The results suggest that reverse bias contributes to the increase of iodine content in SnO_2_ layers or SnO_2_/perovskite interface. It should be ascribed to the directional migration of iodine ions within PSCs under the electric field caused by reverse bias. Also, the shift of peak locations of I 3d and Sn 3d is observed after reverse‐biasing. It further confirms that the migrated iodine ions would interact with SnO_2_, which is similar to the newly formed F—Sn and Cl—Sn bonds.^[^ ^32^, ^33^, ^34^ ^]^ O 1s peaks show the suppressed oxygen vacancies in the SnO_2_ layers after reverse‐biasing, due to formed I—Sn bonds (Figure 3g).^[^ ^33^, ^34^ ^]^ The XPS spectra of other elements including C, N, Br, Cl, Li, and Na have hardly changed after reverse‐biasing (Figures S7 and S8, Supporting Information). XPS spectra of the perovskite layers before and after reverse‐biasing are also measured (Figures S9 and S10, Supporting Information). No obvious changes can be observed in these XPS spectra, indicating that the influence of reverse bias on the upper surface of perovskite and perovskite/spiro‐OMeTAD interface is negligible.

**Figure 4:**
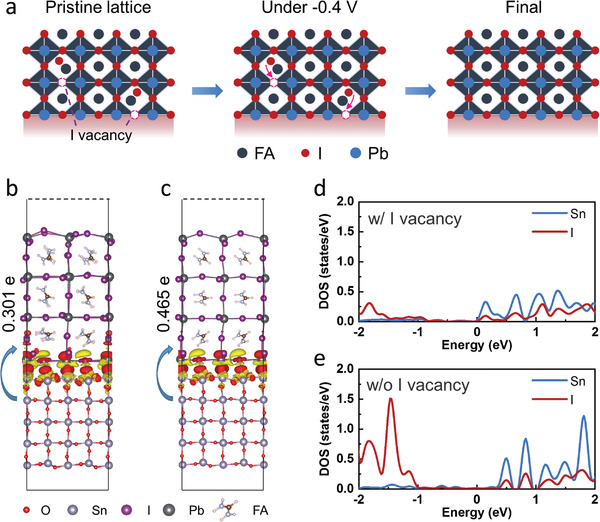
a) Schematic illustration of ion migration within perovskites under reverse bias. Charge density difference coupling with Bader charge analysis b) with and c) without I vacancy. Red represents electron accumulation and yellow represents electron depletion. Density of states of Sn ion and I ion at the SnO_2_/perovskite interface d) with and e) without I vacancy. The Fermi level is set to 0 eV.

It is assumed that iodine ions including iodine interstitials migrate and ultimately accumulate at the SnO_2_/perovskite interface under reverse bias. During this period, parts of these iodine ions will fill the iodine vacancies at the interface, as shown in **Figure** **4**a. Although most of the ions will return to the perovskite bulk after reverse‐biasing, the iodine ions filling the vacancies will be anchored by these traps due to lower energy position. The interaction with SnO_2_ also makes these iodine ions at the interface more stable. It means these ions will stay at the interface and help to reduce interfacial trap‐state density and suppress charge carrier recombination. However, when the PSCs are subjected to heavy reverse voltage, a large number of iodine ions will accumulate at the SnO_2_/perovskite interface, leading to the degradation of perovskite into tiny particles at the interface (Figures S11–S13, Supporting Information). These accumulated ions would weaken the bond bonding between FA cations and I anions within the perovskite lattice, which is deemed to be significant for the stability of the perovskite structure.^[^ ^35^ ^]^ These results may account for the deterioration of device performance after reverse‐biasing at −1.2 and −1.6 V.

The above experimental results confirm the positive effect of reverse‐biasing at −0.4 V. Accordingly, the champion PSC is achieved by using this strategy, in which the efficiency increases from the initial 22.13% (*V*_oc_ = 1.10 V, *J*_sc_ = 24.91 mA cm^−2^, and FF = 0.806) to 23.48% (*V*_oc_ = 1.16 V, *J*_sc_ = 24.85 mA cm^−2^, and FF = 0.816) (Figure S14 and Table S5, Supporting Information). The integrated current density (23.94 mA cm^−2^ and 24.01 mA cm^−2^ for pristine and reverse biased cell) derived from external quantum efficiency (EQE) spectra are consistent with the *J*_sc_ obtained from the *J–V* curves (Figure S15, Supporting Information).

In order to obtain more information about how migrated iodine ions under reverse bias affect the performance of SnO_2_/perovskite interface, first‐principles calculations of SnO_2_/perovskite interfaces with and without I vacancy are performed. The charge transfer at the SnO_2_/perovskite interfaces with and without I vacancy are shown in Figure 4b,c, respectively. Red represents electron accumulation and yellow represents electron depletion. The results reveal that a stronger interaction at the SnO_2_/perovskite interface is obtained. The Bader charge analysis shows that the electrons flow spontaneously from SnO_2_ to perovskite at the interface, which creates a built‐in electric field that points from SnO_2_ to perovskite. A higher charge transfer at the interface without I vacancy (0.465 e) than that at the interface with I vacancy (0.301 e) demonstrates stronger built‐in electric field and better carrier transport capacity at the former interface, which is consistent with experimental results above. The density of states (DOS) of Sn ion and I ion at the SnO_2_/perovskite interfaces with and without I vacancy are shown in Figure 4d,e, respectively. The Fermi level is set to 0 eV. The strong orbital coupling between Sn ion and I ion shows that the Sn—I bonds are formed at the interface, which is consistent with the XPS results, facilitating carrier transport at the interface. The orbital coupling at the interface without I vacancy is stronger than that with I vacancy, which demonstrates better carrier transport capacity.

**Figure 5:**
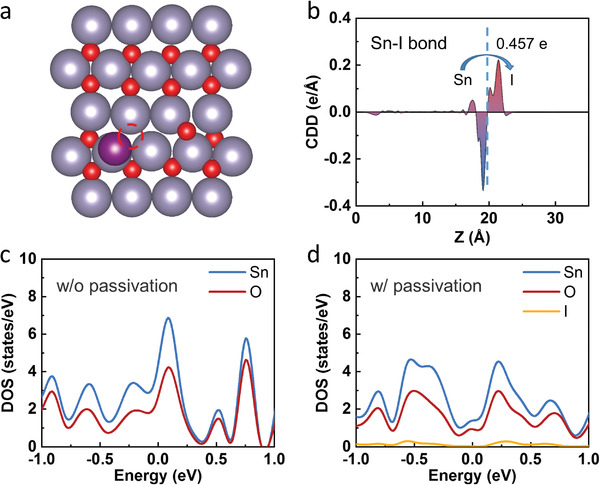
a) Structure model of relaxed SnO_2_ surface with I ion passivating O vacancy. The ions of Sn, O, and I are indicated by gray, red, and purple balls, respectively. The red dotted circle represents O vacancy. b) Plane‐averaged charge density difference along the *Z* direction coupling with Bader charge analysis. The dotted blue line represents the middle of Sn—I bond axis. DOS at the SnO_2_ surface c) without and d) with I ion passivation. The Fermi level is set to 0 eV.

Previous result shows that the iodide could be driven into the electron transport layer.^[^ ^7^ ^]^ Iodine ions may migrate through grain boundaries or amorphous regions of polycrystalline films of perovskite and SnO_2_, which have been recognized as the main channel of ion migration.^[^ ^36^ ^]^ Thus, the mechanism how iodine ions crossing the SnO_2_/perovskite interface affect the performance of SnO_2_ ETL is also explored by using first‐principles calculations. It is found that incorporating an I ion into the SnO_2_ lattice is very difficult and it extremely prefers to stay at the surface of SnO_2_. **Figure** **5**a shows the structure model of relaxed SnO_2_ surface with I ion passivating O vacancy and the I ion is attached to the Sn ion. The plane‐averaged charge density difference along the direction perpendicular to the surface coupling with Bader charge analysis at SnO_2_ surface is calculated, as shown in Figure 5b. The I ion gains a lot of electrons, while the Sn ion loses electrons. It shows that the strong Sn—I ionic bond is formed at the O vacancy. The DOS at the SnO_2_ surface without and with I ion passivation are shown in Figure 5c,d, respectively, and the Fermi level is set to 0 eV. The surface gap states (as the DOS around the Fermi level) with I ion passivation are smaller than those without I ion passivation. Such weakened surface gap states indicate the reduced carrier recombination at the surface region, which is beneficial to improve the PCEs.

It should be pointed out that the performance enhancement induced by reverse‐biasing is not permanent or irreversible. The device performance will return to the initial value after being stored in nitrogen for 23 h, but the performance can be enhanced again by reverse‐biasing after the storage (Figure S16 and Table S6, Supporting Information). It further confirms that the phenomenon found here should be closely related to ion migration. Anyway, the research work shows several implications for the whole perovskite community. i) Special treatment of PSCs, such as extra light soaking or reverse‐biasing, before the scanning of *J–V* curves should be elaborated; ii) ion migration can be utilized to realize the doping or passivation of perovskite, ETL and HTL under external electric field; and iii) the materials containing mobile ions can be used to dope functional films or other materials under external forward or reverse voltage, which could be extended to other research fields such as light emitting diodes, transistors and memristors.

## 3 Conclusion

In summary, the performance of PSCs can be improved by reverse‐biasing and its mechanism has been unveiled. The electric field induced by reverse bias will drive ions to move directionally in perovskite films and accumulate at the interface. Part of accumulated iodine ions will fill the I vacancy at the interface and interact with SnO_2_, even after reverse‐biasing. First‐principles calculations prove that the SnO_2_/perovskite interface without I vacancies has a higher charge transfer and stronger orbital coupling between Sn ions and I ions, resulting in greater built‐in electric field and better carrier transport capacity. When the iodine ions migrate into SnO_2_ layers under reverse bias, the strong Sn—I ionic bond is formed at the O vacancy, leading to reduced surface gap states and suppressed charge carrier recombination on SnO_2_ surface. According to this strategy, a PCE of over 23% was achieved with a simple planar heterojunction structure, indicating obvious enhancement in efficiency from 22.13% for the reference device. The research work not only brings interesting phenomena to perovskite community, but also provides a new idea and route for utilizing the ion migration and improving the photovoltaic performance and stability of PSCs.

## 4 Experimental Section

### Materials

All materials were used directly without any purification: tin oxide precursor (SnO_2_, 15% in H_2_O colloidal dispersion, Alfa Aesar), HC(NH_2_)_2_I (FAI, 99.5%, Xi″an Polymer Light Technology Corp.), [6,6]‐phenyl‐C61‐butyric acid methyl ester (PC_61_BM, Xi″an Polymer Light Technology Corp.), lead iodide (PbI_2_, 99.999%, Sigma‐Aldrich), CH_3_NH_3_Cl (MACl, 99.5%, Xi″an Polymer Light Technology Corp.), CH_3_NH_3_Br (MABr, 99.5%, Xi″an Polymer Light Technology Corp.), cesium iodide (CsI, 99.999%, Sigma‐Aldrich), 2,2’,7,7’‐tetrakis‐(*N,N*‐di(4‐methoxyphenyl)amino)‐ 9,9’‐spirobifluorene (Spiro‐OMeTAD, 99.5%, Xi'an Polymer Light Technology Corp.), isopropanol (99.5%, J&K Scientific), *N,N*‐dimethylformamide (DMF, 99.8%, Sigma‐Aldrich), chlorobenzene (99.8%, Sigma‐Aldrich), dimethyl sulfoxide (DMSO, 99.9%, Sigma‐Aldrich), acetonitrile (99.95%, Sigma‐Aldrich), lithium bis‐(trifluoromethanesulfonyl)imide (Li‐TFSI, 99.95%, Sigma‐Aldrich), and 4‐tert‐butylpyridine (tBP, 98%, Sigma‐Aldrich).

### Device Fabrication

The substrate (glass/ITO) was cleaned by sonication in acetone, detergents/H_2_O, distilled water, and isopropanol for 25 min sequentially, which was then dried by N_2_ blowing and treated by UV‐ozone for 25 min. The SnO_2_ colloidal solution was diluted by H_2_O to 2.67% and filtered by a nylon filter (0.22 µm) before use. For electron transport layer, SnO_2_ precursor solution was spin‐coated at 3000 rpm for 30 s and then annealed at 150 °C for 30 min. The resultant SnO_2_ layers were treated by UV‐ozone for 15 min before the deposition of perovskite films. Note that all of the steps above were carried out under ambient air without controlling temperature and relative humidity of the environment. Then the samples were transferred to a glove box to prepare perovskite films. 1.3 m PbI_2_ and 0.13 m CsI were dissolved in mixed solvent (DMF: DMSO, volume ratio = 0.95: 0.05) and stirred at 75 °C for 8 h. The precursor solution was spin‐coated at a speed of 1500 rpm for 30 s and then annealed at 70 °C for 30 min. The solution of FAI:MABr:MACl (60 mg: 6 mg: 6 mg in 1 mL isopropanol) was spin‐coated at 1500 rpm for 30 s, followed by annealing at 150 °C for 20 min in ambient air (40% relative humidity). Subsequently, the spiro‐OMeTAD solution in chlorobenzene (90 mg mL^−1^), in which 10 µL tBP and 45 µL Li‐TFSI/acetonitrile (170 mg mL^−1^) were added, was spin‐coated at 3500 rpm for 30 s on the perovskite film. Finally, a 100 nm Ag electrode was prepared via thermal evaporation at 8 × 10^−6^ mbar, resulting in an active area of 0.1 cm^2^.

For the fabrication of PSCs with FA*_x_* MA*_y_* Cs_1−_ *_x_*_−_ *_y_* Pb(I*_z_* Cl_1−_ *_z_*)_3_ and FA*_x_* MA_1−_ *_x_* Pb(I*_z_* Br*_k_* Cl_1−_ *_z_*_−_ *_k_*)_3_ perovskite films, MABr, and CsI are removed from the perovskite precursor solution, respectively. For the fabrication of PSCs with poly(3,4‐ethylenedioxythiophene) /poly(styrenesulfonate) (PEDOT: PSS) and PC_61_BM films as the HTL and ETL, respectively, the PEDOT: PSS solution was spin‐coated at 3000 rpm for 30 s and then annealed at 150 °C for 15 min, and the PC_61_BM solution (15 mg mL^−1^ in chlorobenzene) was spin‐coated onto perovskite films at a speed of 3000 rpm for 30 s. For the fabrication of MAPbI_3_ layer, the perovskite precursor solution with a concentration of 550 mg mL^−1^ (molar ratio between CH_3_NH_3_I and PbI_2_ is 1: 1) was spin‐coated at 4000 rpm for 40 s, during which 65 µL chlorobenzene was dropped to improve the crystallization process. Subsequently, the deposited perovskite layer was annealed at 100 °C for 10 min. For the fabrication of PC_61_BM modification layer between SnO_2_ and perovskite, the PC_61_BM solution (10 mg mL^−1^ in chlorobenzene) was spin‐coated onto SnO_2_ film at 2000 rpm for 30 s and then annealed at 100 °C for 10 min in a glove box. The carbon electrode was prepared by press transfer technique reported in the literature.^[^ ^37^ ^]^

### Reverse‐Biasing Experiment

In order to obtain the improvement in device performance, the PSCs were reverse biased for several minutes in dark. Note that the PSCs should be self‐stable under ambient air and under repeating *J–V* scanning. After reverse‐biasing, the device performance cannot be improved directly because the ions accumulated at the interface need to migrate back to their own sites. Therefore, multiple *J–V* scanning are needed. During this period, the device performance increases gradually and finally stabilizes at a higher value than the initial efficiency (Figure S17 and Table S7, Supporting Information). The whole process was carried out under ambient air (≈25 °C) with controlled relative humidity of ≈40%.

The perovskite and SnO_2_ films that are used for characterizations, including SEM, XRD, absorption spectra, and XPS, are derived from the PSCs with and without reverse‐biasing. Specifically, the silver electrode and spiro‐OMeTAD layer are removed by the Scotch type and spin‐coating of chlorobenzene for three times, respectively. To expose underneath SnO_2_ layers, DMF was subsequently spin‐coated on the samples for three times.

### Characterization

The absorption properties of perovskite films were measured by UV–vis spectrophotometer (Puxi, T9, China). X‐ray diffraction (Rigaku D, Max 2500, Japan) was employed to characterize the crystallographic properties of perovskite. Scanning electron microscope (FEI Helios Nanolab 600i SEM) was utilized to characterize the surface morphology of perovskite films and cross section of the PSC. EIS (CHI606D, Chenhua, Shanghai) was employed to investigate the electrical dynamics of PSCs. XPS (Al K*α* X‐ray source, 1486.6 eV) was used to characterize elemental changes of perovskite and SnO_2_ films. Capacitance–voltage (*C*–*V*) curves were measured under dark at a frequency of 5 kHz. Transient photovoltage (Keysight DSO‐X 3104A, NL100, 500 nm) decay and photocurrent (Keysight DSO‐X 3104A, 1 MW and 50 W) decay were utilized to investigate the carrier transport and recombination properties. The EQE spectra were measured using a Saifan (Beijing) EQE measurement system. *J–V* curves of the cells were measured from +1.2 V to 0 V (50 ms scanning delay, 60 points, about 100 mV s^−1^ scan rate) by a digital Source Meter (Keithley, model 2420). A Xenon‐lamp‐based solar simulator (Newport 91160s, air mass 1.5 ) with a light intensity of 100 mW cm^−2^ was calibrated by a standard silicon solar cell before the measurements.

### Computational Methods

The first‐principles calculations were performed using the Vienna ab initio simulation package based on density functional theory with generalized gradient approximation of a Perdew–Burke–Ernzerhof functional.^[^ ^38^, ^39^ ^]^ Valence Core interactions were described by projector‐augmented‐wave pseudopotentials.^[^ ^40^ ^]^ The simulations used 350 eV plane‐wave energy cutoff and Gamma point representing the Brillouin zone integrations. All Atomic positions were fully relaxed, until the total force on each atom was less than 0.03 eV Å^−1^ and the total energy change was less than 1×10^−4^ eV. A 4×2 supercell of (110)‐plane rutile SnO_2_ and a 2×2 supercell of (001)‐plane cubic FAPbI_3_ surfaces were used. A vacuum space was set to about 15 Å in the *Z* direction to minimize the artificial interlayer interactions.

### Statistical Analysis

No preprocessing was used for data of statistical analysis. Data presentation was exhibited as the mean ± standard deviation. Sample size (*n*) was shown in the corresponding table. Statistical analysis was performed using Origin 2021.

## Conflict of Interest

The authors declare no conflict of interest.

## Supporting information

Supporting Information

## Data Availability

The data that support the findings of this study are available from the corresponding author upon reasonable request.
