# Supplementary material for: Manipulating the Migration of Iodine Ions via Reverse‐Biasing for Boosting Photovoltaic Performance of Perovskite Solar Cells
Source: Adv Sci (Weinh). 2022 Oct 26;9(35):2204163. doi: 10.1002/advs.202204163 (PMC9762299; doi:10.1002/advs.202204163)
Supplement: Supplementary file 1 — Supporting Information [file ADVS-9-2204163-s001.pdf]

ADVANCED SCIENCE
Open Access

Supporting Information

for *Adv. Sci.*, DOI 10.1002/advs.202204163

Manipulating the Migration of Iodine Ions via Reverse-Biasing for Boosting Photovoltaic Performance of Perovskite Solar Cells

Keqing Huang, Xiangxiang Feng, Hengyue Li, Caoyu Long, Biao Liu, Jiangjian Shi, Qingbo Meng, Klaus Weber, The Duong and Junliang Yang**

Supporting Information

Manipulating the Migration of Iodine Ions via Reverse-Biasing for Boosting Photovoltaic Performance of Perovskite Solar Cells

Keqing Huang,[†] Xiangxiang Feng,[†] Hengyue Li, Caoyu Long, Biao Liu, Jiangjian Shi, Qingbo Meng, Klaus Weber, The Duong, Junliang Yang**

K. Huang, X. Feng, H. Li, C. Long, Dr. B. Liu, Prof. J. Yang
Hunan Key Laboratory for Super-microstructure and Ultrafast Process, School of Physics and Electronics
Central South University
Changsha 410083, China
E-mail: junliang.yang@csu.edu.cn

K. Huang, Dr. T. Duong, Prof. K. Weber
College of Engineering and Computer Science
Australian National University
Canberra Australian Capital Territory 2600, Australia
E-mail: the.duong@anu.edu.au

Dr. J. Shi, Prof. Q. Meng
Key Laboratory for Renewable Energy
Chinese Academy of Sciences
Beijing Key Laboratory for New Energy Materials and Devices
Institute of Physics
Chinese Academy of Sciences
Beijing 100190, P. R. China

[†] These authors contributed equally to this work.

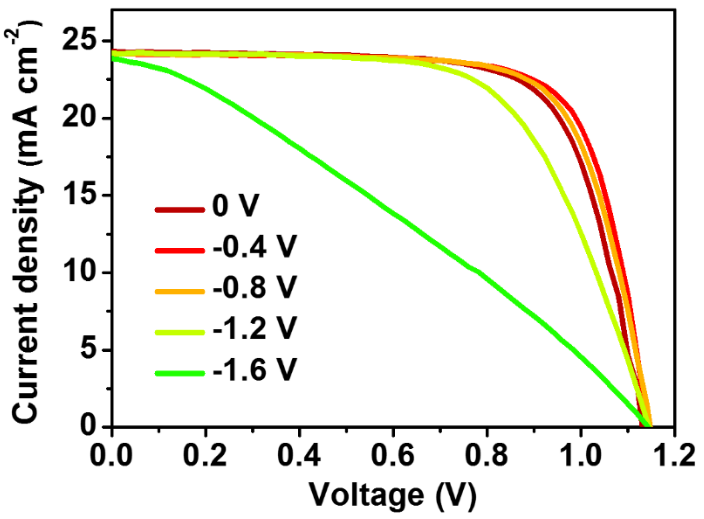

Figure S1. Forward scanned *J*-*V* curves of a typical PSC after reverse-biasing at different voltage for 3 min.

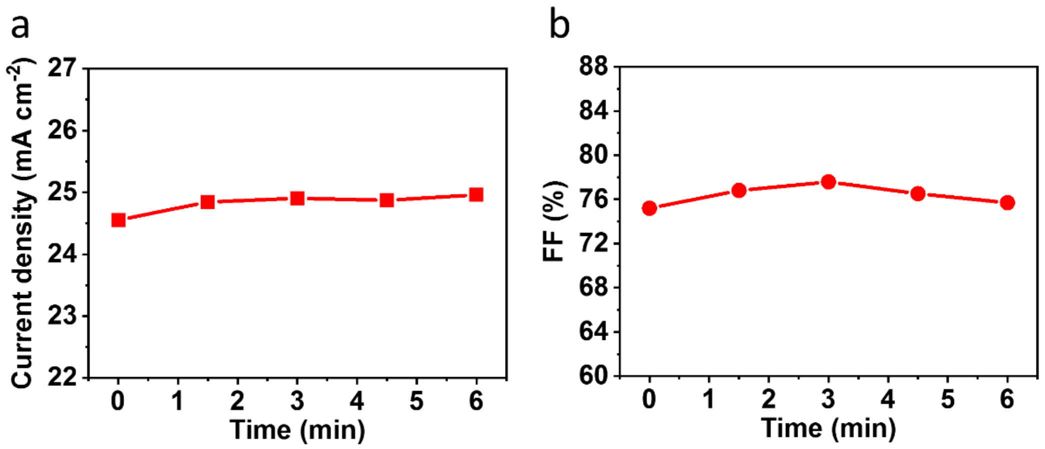

Figure S2. (a) J_{sc} and (b) FF of the PSC after reverse-biasing at -0.4 V for different time.

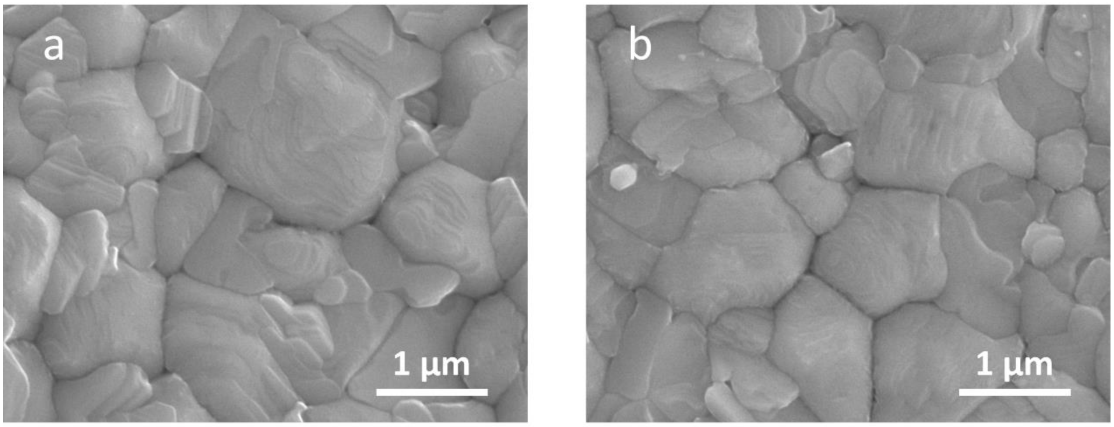

Figure S3. SEM images of the perovskite films (a) without and (b) with reverse-biasing at -0.4 V for 3 min. Note that the perovskite films are derived from the PSCs with and without reverse-biasing. Specifically, the silver electrode and spiro-OMeTAD layer are removed by the Scotch type and washing with chlorobenzene, respectively.

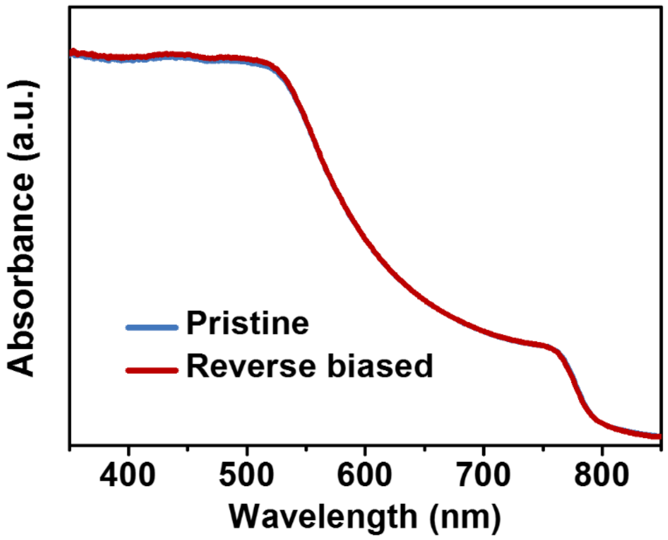

Figure S4. (a) UV–vis absorption spectra of the perovskite films without and with reverse-biasing at -0.4 V for 3 min.

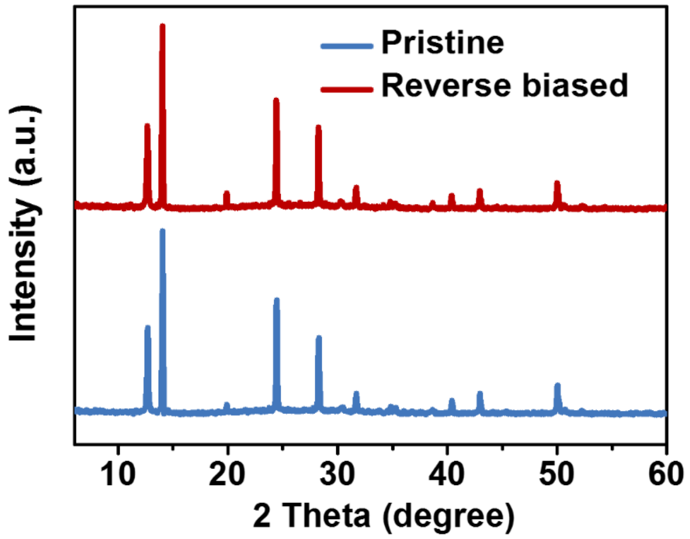

Figure S5. XRD patterns of the perovskite films without and with reverse-biasing at -0.4 V for 3 min.

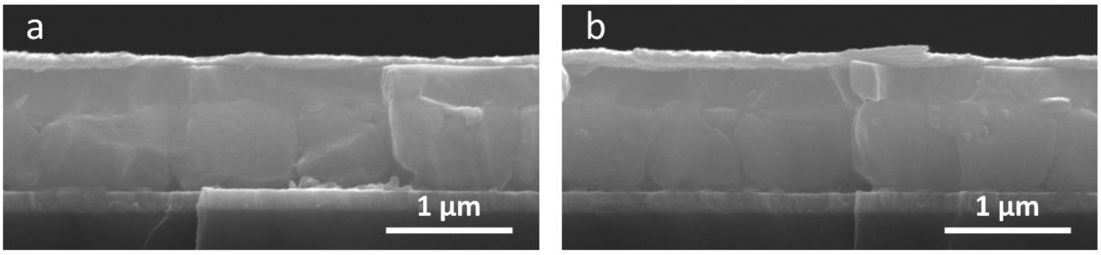

Figure S6. SEM cross-section images of PSCs (a) without and (b) with reverse-biasing at -0.4 V for 3 min.

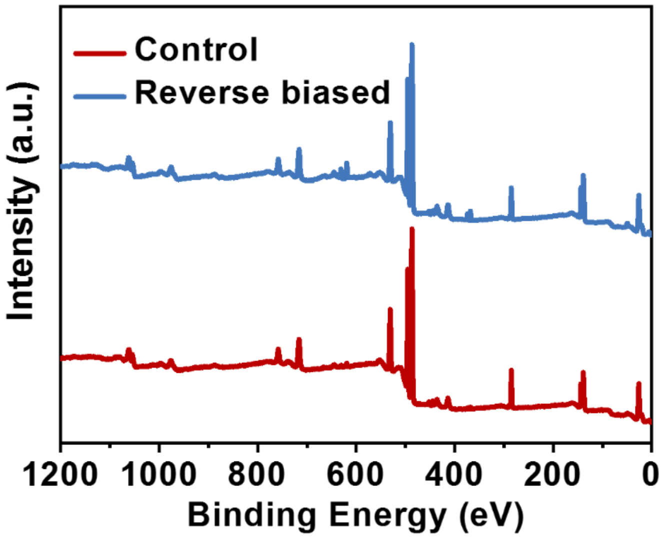

Figure S7. XPS spectra of SnO_2 layers without and with reverse-biasing at -0.4 V for 3 min.

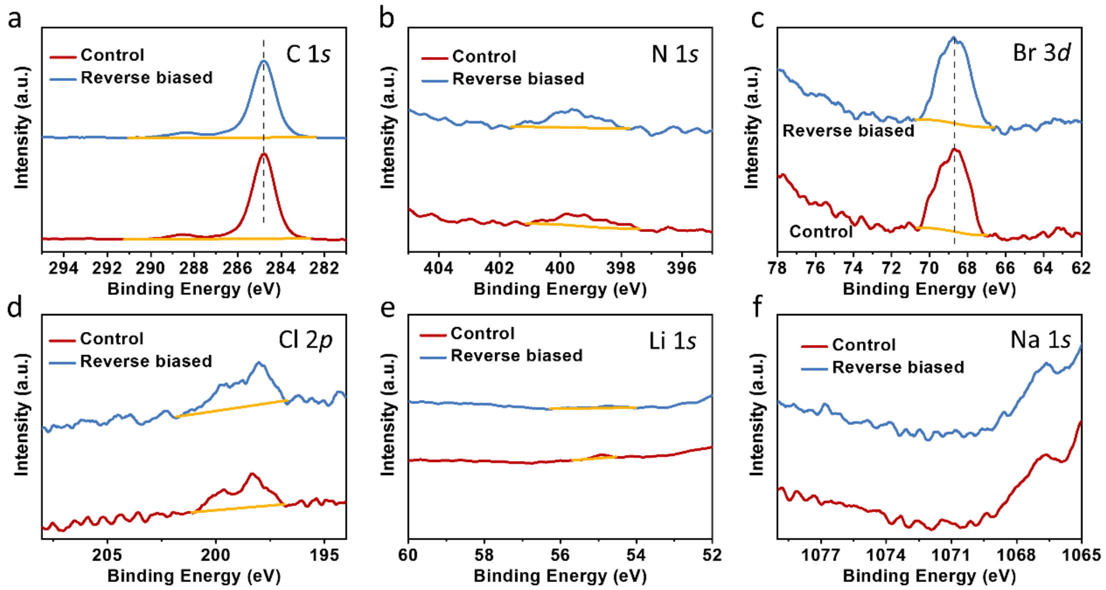

Figure S8. XPS spectra for (a) C, (b) N, (c) Br, (d) Cl, (e) Li and (f) Na elements of SnO_2 layers without and with reverse-biasing at -0.4 V for 3 min.

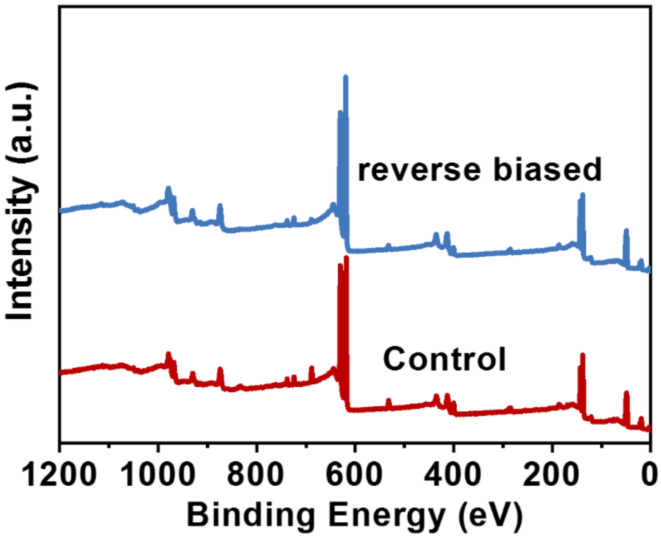

Figure S9. XPS spectra of perovskite layers without and with reverse-biasing at -0.4 V for 3 min.

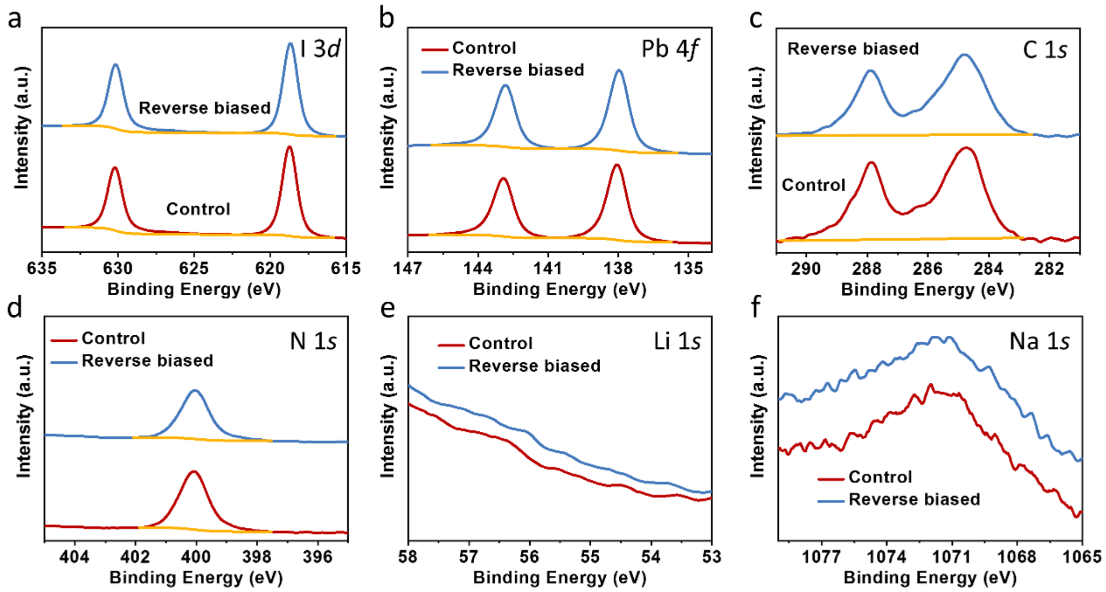

Figure S10. XPS spectra for (a) I, (b) Pb, (c) C, (d) N, (e) Li and (f) Na elements of perovskite layers without and with reverse-biasing at -0.4 V for 3 min.

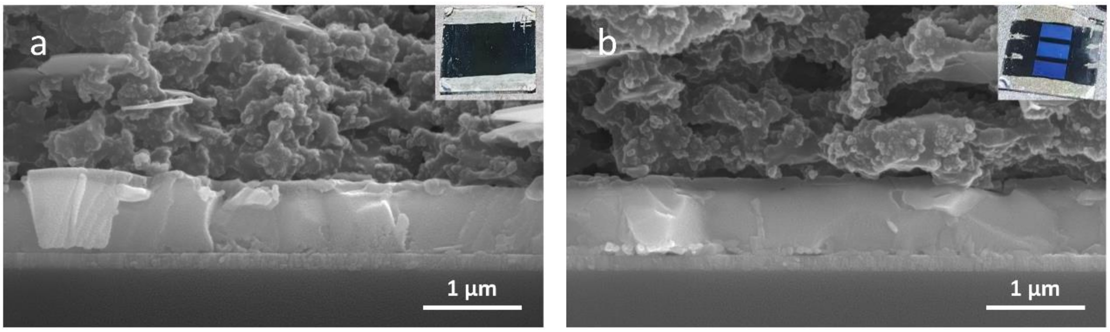

Figure S11. SEM cross-section images of the PSCs (a) without and (b) with reverse-biasing under constant reverse current density of -10 mA cm^{-2} for 3 min. Insets in (a) and (b) show the optical images of the PSCs without and with reverse-biasing, respectively. Note that cell configuration of ITO/SnO_2/$FA_{0.945}MA_{0.025}Cs_{0.03}Pb(I_{0.975}Br_{0.025})_3$/Spiro-OMeTAD/Carbon is adopted to avoid the effect of Ag electrode during reverse-biasing. Also, the thickness of spiro-OMeTAD is decreased to obtain better device performance.

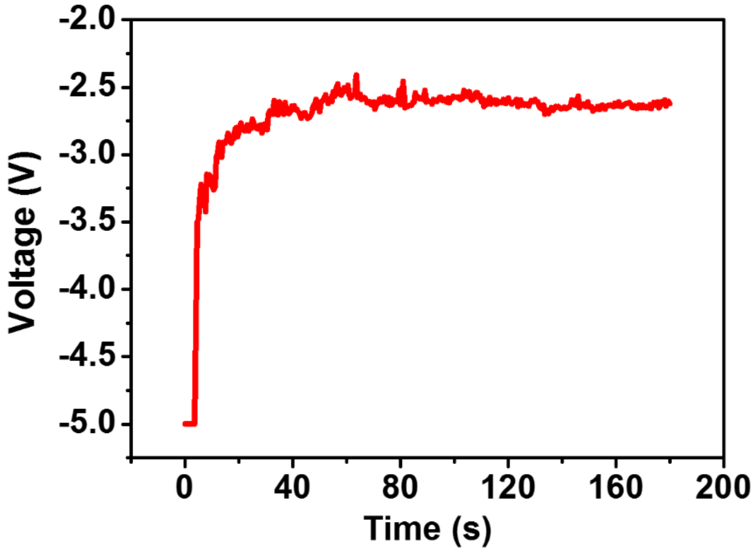

Figure S12. Voltage of the PSCs under constant reverse current density of -10 mA cm^{-2} for 3 min. The limiting voltage is 5 V.

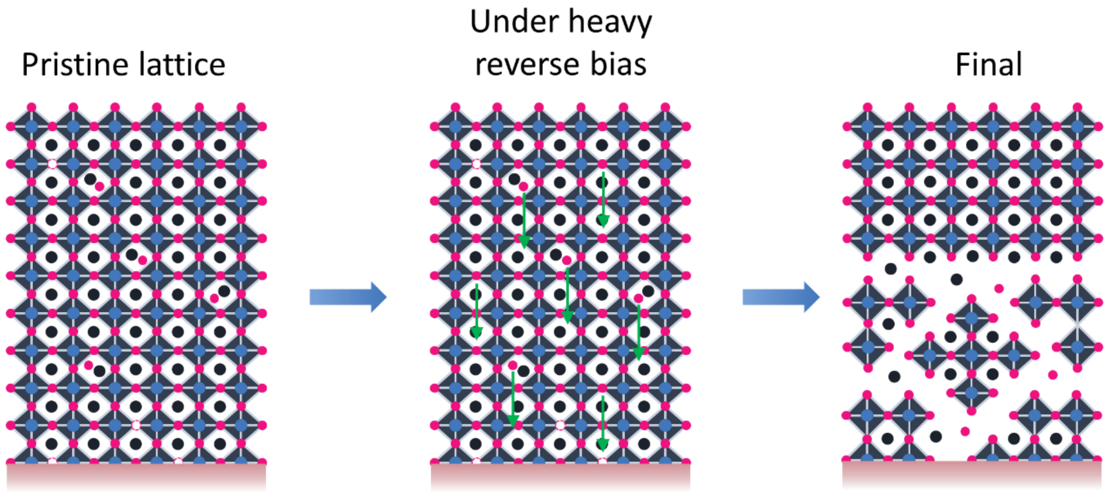

Figure S13. Schematic illustration of the evolution of perovskites under heavy reverse bias.

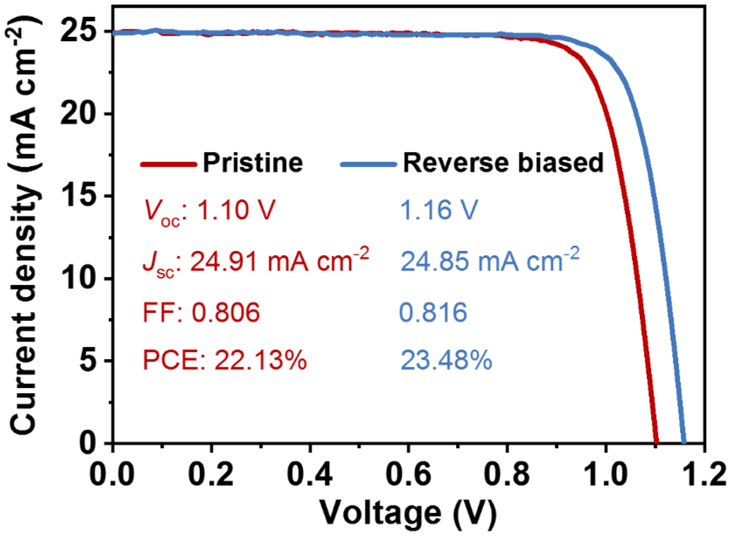

Figure S14. *J*-*V* curves of the PSC before and after reverse-biasing at -0.4 V for 3 min.

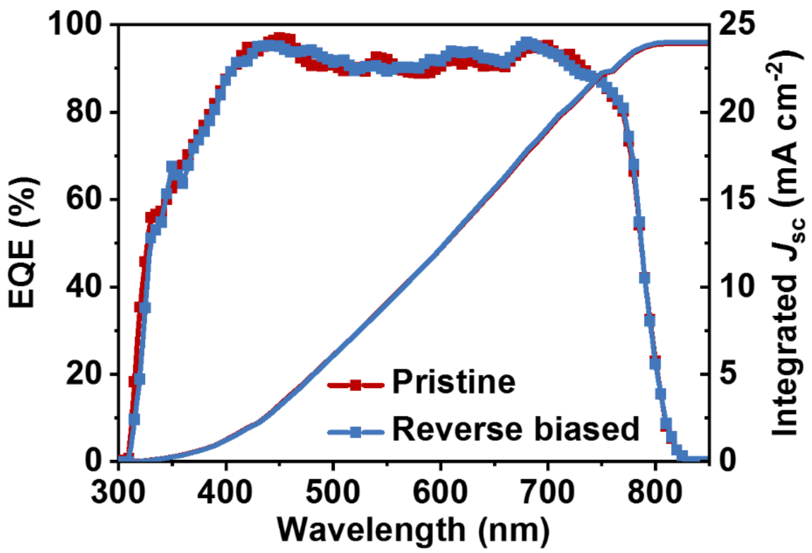

Figure S15. EQE spectra of the PSC before and after reverse-biasing at -0.4 V for 3 min.

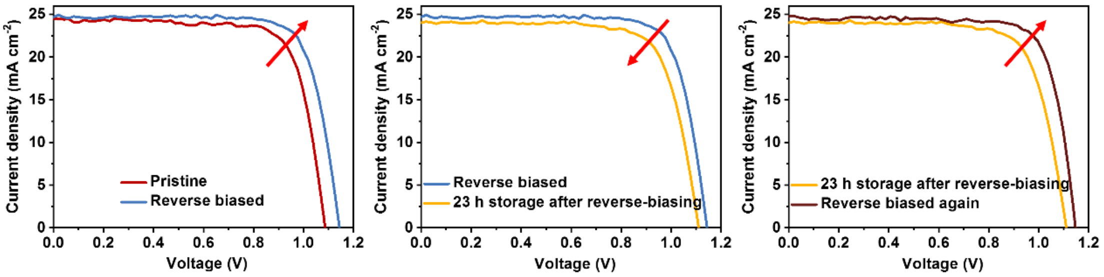

Figure S16. Evolution of *J*-*V* curves of the PSC after reverse-biasing and storage in nitrogen.

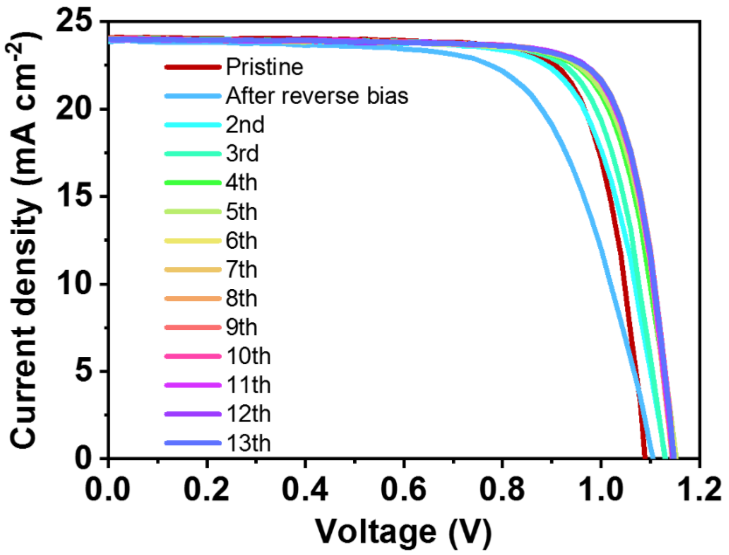

Figure S17. *J*-*V* curves for the reverse biased PSC under repeating tests.

Table S1. Performance parameters of the PSC after reverse-biasing at -0.4 V for the different time.

Time (min)	V_{oc} (V)	J_{sc} (mA cm^{-2})	FF	PCE (%)
0	1.10	24.55	0.752	20.40
1.5	1.14	24.84	0.768	21.71
3	1.14	24.90	0.776	22.11
4.5	1.15	24.87	0.765	21.83
6	1.15	24.96	0.757	21.72

Table S2. Performance parameters for PSCs with different structure before and after reverse-biasing at -0.4 V for 3 min. Each result with a specific cell configuration is derived from at least three PSCs.

Cell configuration	Δ V_{oc} (V)[a)]	Δ J_{sc} (mA cm^{-2})[b)]	Δ FF[c)]	Δ PCE (%)[d)]
ITO/SnO_2/$FA_xMA_yCs_{1-x-y}Pb(I_zBr_kCl_{1-z-k})_3$/spiro-OMeTAD/Ag	0.03	0.01	0.016	1.03
ITO/SnO_2/$FA_xMA_yCs_{1-x-y}Pb(I_zCl_{1-z})_3$/spiro-OMeTAD/Ag	0.03	-0.34	0.007	0.5
ITO/SnO_2/$FA_xMA_{1-x}Pb(I_zBr_kCl_{1-z-k})_3$/spiro-OMeTAD/Ag	0.06	0.28	0.02	1.67
ITO/PEDOT: PSS/$FA_xMA_yCs_{1-x-y}Pb(I_zBr_kCl_{1-z-k})_3$/$PC_{61}BM$/Ag	-0.55	0.073	-0.102	-3.64
ITO/PEDOT: PSS/$MAPbI_3$/$PC_{61}BM$/Ag	-0.34	-2.69	-0.131	-3.67
ITO/SnO_2/$PC_{61}BM$/$FA_xMA_yCs_{1-x-y}Pb(I_zBr_kCl_{1-z-k})_3$/spiro-OMeTAD/Ag	-0.02	-0.13	-0.014	-0.84
ITO/$FA_xMA_yCs_{1-x-y}Pb(I_zBr_kCl_{1-z-k})_3$/spiro-OMeTAD/Ag	-0.02	-0.33	-0.012	-0.34
ITO/$FA_xMA_yCs_{1-x-y}Pb(I_zCl_{1-z})_3$/spiro-OMeTAD/Ag	-0.02	-0.39	-0.024	-0.84
ITO/$FA_xMA_{1-x}Pb(I_zBr_kCl_{1-z-k})_3$/spiro-OMeTAD/Ag	-0.04	-0.29	-0.031	-1.00
ITO/SnO_2/$FA_xMA_yCs_{1-x-y}Pb(I_zBr_kCl_{1-z-k})_3$/Carbon	0.03	0.83	0.049	1.35

a) Δ V_{oc}= $V_{oc\ pristine}$ – $V_{oc\ reverse\ biased}$

b) Δ J_{sc} = $J_{sc\ pristine}$ – $J_{sc\ reverse\ biased}$

c) Δ FF= $FF_{pristine}$ – $FF_{reverse\ biased}$

d) Δ PCE = $PCE_{pristine}$ – $PCE_{reverse\ biased}$

Table S3. The fitted parameters of *I*-*t* curves of the PSCs under -0.4 V and different temperature.

Temperature (^{o}C)	I_0	I_1	k_1	I_2	k_2
25	0.003	0.650	0.378	0.344	5.508
32.5	0.005	0.677	0.530	0.313	4.360
40	0.001	0.708	0.377	0.289	3.245
47.5	0.005	0.717	0.003	0.278	2.249
55	0.004	0.683	0.003	0.313	1.771

Table S4. Activation energy of different type of ions in perovskites.

Ion	Perovskite	Activation energy (eV)	Reference
I^-	$MAPbI_3$	0.16	1
I^-	$MAPbI_xCl_y$	0.23	1
I^-	$MAPbI_3$	0.32	1
I^-	$MAPbI_3$	0.58	1
I^-	$MAPbI_3$	0.43	1
MA^+	$MAPbI_3$	0.36	1
MA^+	$MAPbI_3$	0.43	1
V_I	$MAPbI_3$	0.318	2
V_I	$MAPbI_3$	0.316	2
V_I	$MAPbI_3$	0.44	2
V_I	$MAPbI_3$	0.418	2
V_I	$FAPbI_3$	0.548	2
V_I	$FAPbI_3$	0.555	2
V_I	$FAPbI_3$	0.477	2
V_I	$FAPbI_3$	0.474	2
V_I^+	$MAPbI_3$	0.332	2
V_I^+	$MAPbI_3$	0.454	2
V_I^+	$FAPbI_3$	0.501	2
V_I^+	$FAPbI_3$	0.424	2
V_{MA}	$MAPbI_3$	0.57	2
V_{MA}	$MAPbI_3$	0.567	2
V_{MA}	$MAPbI_3$	0.894	2
V_{MA}	$MAPbI_3$	0.877	2
V_{MA}	$MAPbI_3$	0.46	3
V_{MA}	$MAPbBr_3$	0.56	3
V_{MA}^-	$MAPbI_3$	0.548	2
V_{MA}^-	$MAPbI_3$	0.888	2

V_{FA}	$FAPbI_3$	0.611	2
V_{FA}	$FAPbI_3$	0.582	2
V_{FA}	$FAPbI_3$	0.61	2
V_{FA}	$FAPbI_3$	0.612	2
V_{FA}^-	$FAPbI_3$	0.571	2
V_{FA}^-	$FAPbI_3$	0.592	2
V_{Pb}	$MAPbI_3$	0.8	3
I_{Cl}	$MAPbI_3$	0.316	2
I_{Cl}	$MAPbI_3$	0.322	2
I_{Cl}^+	$MAPbI_3$	0.32	2
I_i	$MAPbI_3$	0.16	3

Table S5. Performance parameters of the champion PSC before and after reverse-biasing at -0.4 V for 3 min.

Reverse bias	V_{oc} (V)	J_{sc} (mA cm^{-2})	FF	PCE (%)
w/o	1.10	24.91	0.806	22.13
w/	1.16	24.85	0.816	23.48

Table S6. Performance evolution of the PSC after reverse-biasing and storage in nitrogen.

Conditions	V_{oc} (V)	J_{sc} (mA cm^{-2})	FF	PCE (%)
Pristine	1.09	24.35	0.759	20.07
Reverse biased	1.14	24.70	0.774	21.85
23 h storage after reverse-biasing	1.11	24.07	0.743	19.86
Reverse biased again after the storage	1.15	24.58	0.781	21.99

Table S7. Performance parameters for the reverse biased PSC under repeating tests.

Scan	V_{oc} (V)	J_{sc} (mA cm^{-2})	FF	PCE (%)
Pristine	1.09	24.05	0.777	20.34
After reverse bias	1.11	23.85	0.677	17.87
2 times	1.13	23.94	0.740	20.03
3 times	1.13	23.97	0.767	20.77
4 times	1.15	24.01	0.773	21.31
5 times	1.15	23.99	0.775	21.42
6 times	1.15	24.02	0.785	21.67
7 times	1.14	24.01	0.789	21.65
8 times	1.14	24.05	0.790	21.74
9 times	1.14	24.00	0.791	21.71
10 times	1.14	24.00	0.790	21.72
11 times	1.14	23.99	0.790	21.70
12 times	1.14	23.99	0.792	21.73
13 times	1.15	24.00	0.788	21.71

References

[1] S. Bae, S. Kim, S.-W. Lee, K. J. Cho, S. Park, S. Lee, Y. Kang, H.-S. Lee, D. Kim, *J. Phys. Chem. Lett*. **2016**, *7*, 3091.

[2] J. Haruyama, K. Sodeyama, L. Han, Y. Tateyama, *J. Am. Chem. Soc*. **2015**, *137*, 10048.

[3] J. M. Azpiroz, E. Mosconi, J. Bisquert, F. D. Angelis, *Energy Environ. Sci*. **2015**, *8*, 2118.
